# Compare Two Kinds of Recurrent MI-Arrest Oocytes

**DOI:** 10.1007/s13224-023-01817-0

**Published:** 2023-08-24

**Authors:** Yan Jiang, Jing-chuan Yuan, Ge Song, Xiao-hua Wu

**Affiliations:** The Center for Reproductive Medicine and Infertility, The Fourth Hospital of Shijiazhuang, Shijiazhuang Obstetrics and Gynecology Hospital affiliated to Hebei Medical University, Hebei Key Laboratory of Maternal and Fetal Medicine, Shijiazhuang, 050011 Hebei People’s Republic of China

**Keywords:** ICSI, Re-ICSI, Recurrent MI-arrest oocytes, Indented ZP

## Introduction

During the past few decades, assisted reproductive technology (ART) is widely practiced throughout the world. The purpose of ovarian stimulation in IVF is to recover mature oocytes at metaphase II (MII) stage which are capable of fertilization either when mixed with sperm or after intracytoplasmic sperm injection (ICSI). However, It is common to obtain metaphase I (MI) or germinal vesicle (GV) oocytes in in vitro fertilization (IVF) after controlled ovarian stimulation (COS) [1]. The immature metaphase I oocytes showed a more structured zona pellucida with smooth or blebbing cumulus cells [2]. Two basic ZP layers—outer with rough spongy appearance and inner with smaller fenestrations and smooth fibrous network—were visible.

The cases previously reported recurrent all MI oocytes were resistant to mature after in vitro maturation (IVM) and no fertilization after ICSI [1, 3–5]. The zona pellucida (ZP) of these MI mature-resistant oocytes was observed to be immature, narrow and fibrous [3].

Other studies reported that all oocytes recurrent presented indented ZP with narrow perivitelline space and significantly low oocyte maturity [6, 7]. Sousa [7] reported that all oocytes from one patient were MI oocytes with indented ZP in two cycles.

The aim of the present study was to compare two types of recurrent MI oocytes characterized by narrow perivitelline space according to ZP, and to evaluate the fertilization, embryological characteristics of all MI oocytes after ICSI.

## Patients and Methods

### Patients

**Fig. 1:**
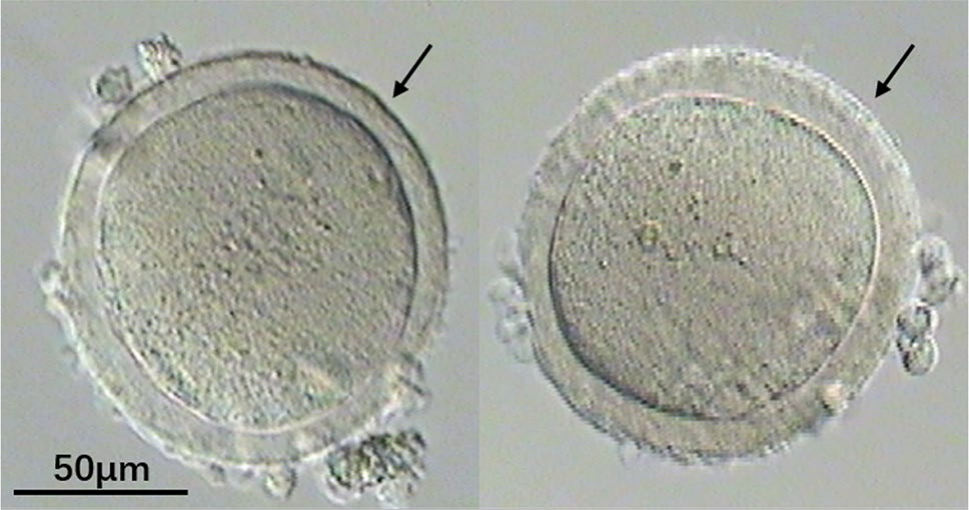
MI oocytes with smooth ZP

**Fig. 2:**
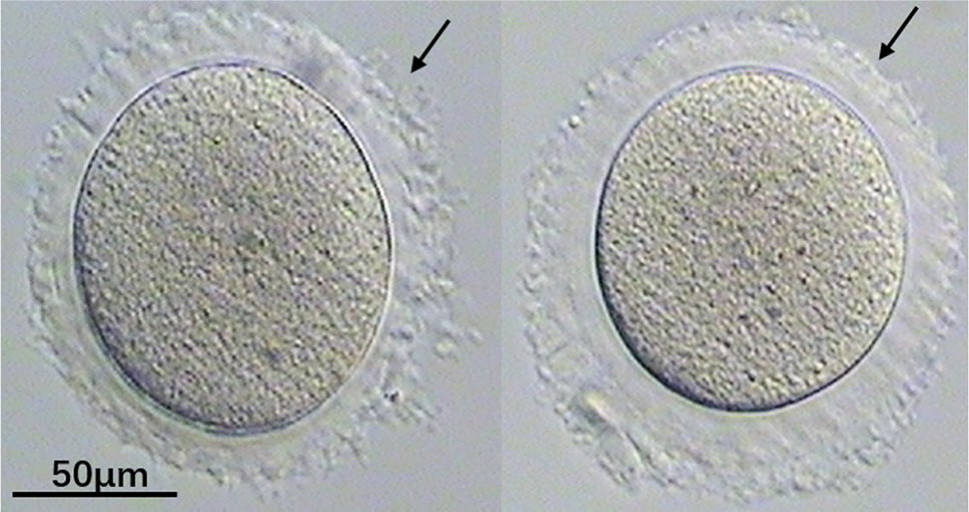
MI oocytes with indented ZP

This was a retrospective study carried out at the Center for Reproductive Medicine and Infertility, The Fourth Hospital of Shijiazhuang in China from March 2017 to May 2020. There were three cases in five cycles with all MI oocytes retrieved divided into two groups according to ZP. In the first case, all MI oocytes with immature and smooth ZP in two cycles were smooth ZP group (Fig. 1). In the other two cases, all MI oocytes with indented ZP were indented ZP group (Fig. 2). The Fourth Hospital of Shijiazhuang ethics committee approved this study (ethical approval number: 20210081).

### Stimulation Protocol and Embryo Culture

Women underwent controlled ovarian hyperstimulation using GnRH short antagonist protocol, or long GnRH agonist protocol. For stimulation, follicle stimulating hormone (FSH) was used (Puregon; Organon; Gonal-F; Merck Serono) combined with human menopausal gonadotropin (HMG; Ferring, Kiel, Germany). The FSH dosage was 150–225 IU. Stimulation timing was from 8 to 11 days. For triggering final oocyte maturation, human chorionic gonadotrophin (HCG) was administered (10,000 IU) (Pregnyl; Organon). Spermatozoa preparation: semen was performed by density gradient centrifugation if the sperm was normal. The semen was washed by centrifugation or slight density gradient centrifugation for ICSI sperm.

The women were given short-time IVF protocol in the first ART treatment cycle unless accompanied with severe male-factor infertility. Oocytes were inseminated with prepared spermatozoa in 2 to 4 h after oocytes retrieval and the cumulus cells were removed mechanically after 4–5 h co-incubation for polar body observation to decide whether to perform rescue ICSI (Re-ICSI) [8].

Traditional ICSI treatment was performed if the number of forward movement sperm collected from the husband was less than 5 million on the day of insemination. Another reason for ICSI decision was for experienced fertilization failure in previous IVF cycles. All MI oocytes preformed ICSI/Re-ICSI cycles.

Embryos were cultured in G-1 (Vitrolife, Sweden) culture medium with 6.0% CO_2_ at 37℃. Normal fertilization was assessed at 16 to 20 h (day 1) after insemination by the appearance of two pronuclei (2PN). High-quality (HQ) embryos were defined as having 7–9 cells on day 3, less than 20% fragments, be a little uneven in appearance. Blastocysts with a score ≥ 3, including those with grades BC and CB, were selected on D5 or D6. Rates of fertilization, cleavage, HQ embryo and blastocyst were compared between the two groups.

The participant gave written informed consent, and the Fourth Hospital of Shijiazhuang ethics committee approved this study (ethical approval number: 20210081).

### Statistical Analysis

Analyses were performed using the SPSS 13.0 statistical software package (SPSS Inc., Chicago, IL, USA). Rate differences between the groups were analyzed using *χ*2 tests and *P* < 0.05 was considered statistically significant.

## Results

**Fig. 3:**
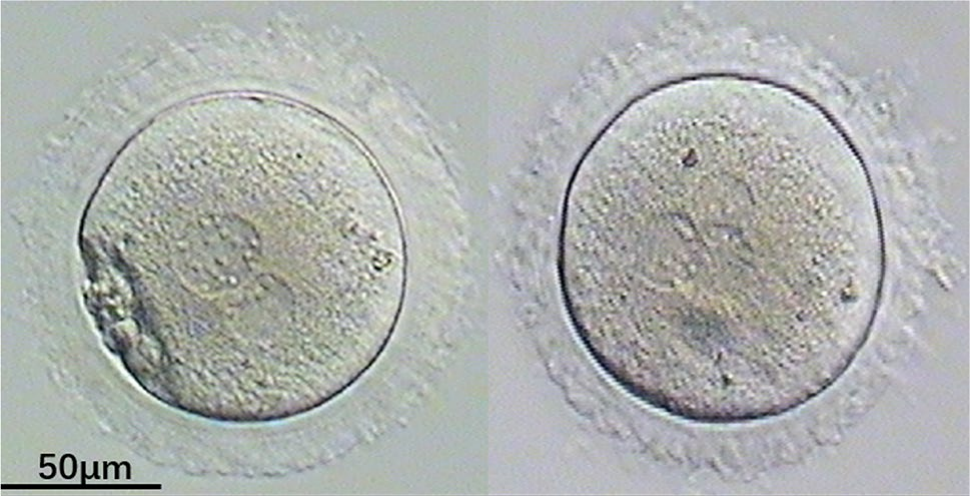
2PN/3PN oocytes with indented ZP

**Fig. 4:**
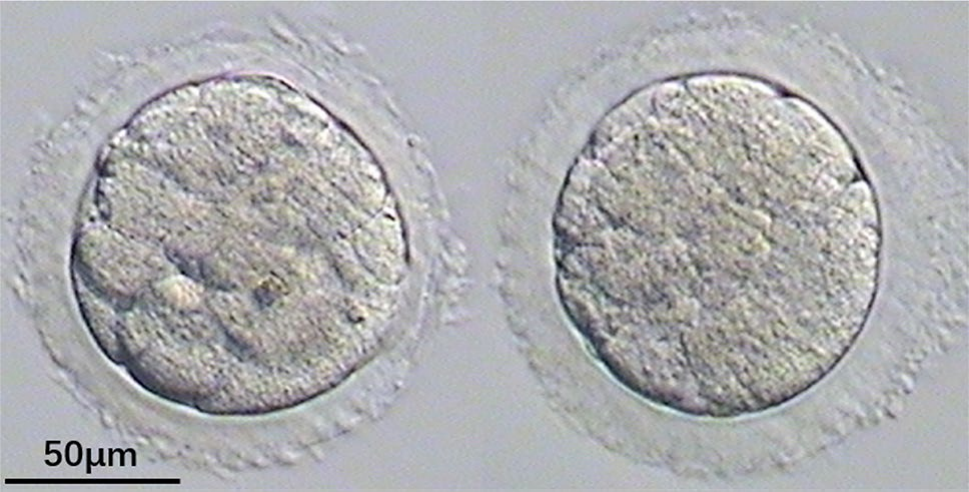
embryo with indented ZP

Total three cases in five cycles with all MI oocytes retrieved. In case one, all MI oocytes were smooth ZP with no fertilization and embryo in two ICSI/Re-ICSI cycles. The other two patients were all MI oocytes with indented ZP. MI oocytes with indented ZP can be normally fertilized and develop blastocyst after ICSI/Re-ICSI. In case 2, embryo transferred and got pregnant with laser-assisted hatching (AH) in the second cycle (Table 1) (Figs. 3, 4).

**Table 1:** Clinical and oocyte characteristics of all MI oocytes cycles in three patients

P	Cycles	Age-F	Age-M	MI oocytes	ZP	ICSI/Re-ICSI Deg	2PN	HQ	Embryos	Blastocyst	Outcome
1	1st	28	30	5	Smooth	Re-ICSI	2	0	0	0	No ET
1	2nd	29	30	14	Smooth	ICSI	3	0	0	0	No ET
2	1st	26	27	10	Indented	Re-ICSI	1	3	1	1	No pregnancy
2	2nd	26	27	14	Indented	ICSI	1	5	0	1	Pregnancy
3	1st	30	33	6	Indented	ICSI	1	1	0	0	No ET

There were no fertilization and embryo after ICSI/Re-ICSI in smooth ZP group. The fertilization rate, 2PN rate and cleavage rate of indented ZP group were significantly higher than those of smooth ZP group (*P* < 0.05) (Table 2).

**Table 2:** Comparison of the two kinds of recurrent MI oocytes for ICSI/Re-ICSI

	Smooth ZP group	Indented ZP group	*χ*^2^	*P* 值
Cycles	2	3		
MI oocytes	19	30		
Fertilization (%)	0/19(0%)	14/30(46.7%)	12.413	0.002*
1PN (%)	0/19(0%)	3/30(10.0%)	2.024	0.198
2PN (%)	0/19(0%)	9/30(30.0%)	6.983	0.007*
Multi-PN (%)	0/19(0%)	2/30(6.7%)	1.321	0.245
Deg (%)	5/19(26.3%)	3/30(10.0%)	2.267	0.132
2PN cleavage (%)	0/13(0%)	8/9(88.9%)	18.159	0.001*
HQ embryos (%)	0/11(0%)	1/8(12.5%)	1.451	0.198
Blastocyst (%)	0/11(0%)	2/8(25.0%)	3.074	0.085

*P < 0.05

### Summary

Distinguishing the two types of MI-arrest oocytes according to ZP is useful for predicting the outcome of recurrent MI oocytes.

## Discussion

In IVF, there were about 20% immature oocytes [3]. The MI oocytes from patients with a normal number of MII oocytes can be normally fertilized and pregnancy after ICSI [9, 10]. These MI oocytes performed ICSI on the day of oocyte aspiration resulting in lower fertilization rates, but blastocyst development rate was significantly higher compared with MI-MII after IVM [11]. The MI oocyte can complete the extrusion of its PB after ICSI in human [9]. A higher proportion of MI oocytes progressed to MII after fertilization in mouse oocytes [12].

However, all oocytes were MI arrest in repeated IVF treatment that is extremely rare [13, 14]. There may be unidentified in vivo factors on the oocyte maturation causing low developmental capacity [15, 16].

In this study, two patients were all MI oocytes in repeated cycles. The reason of repeated all MI arrest is unknown, underwent IVM and ICSI without further maturation or fertilization [17]. Currently, several genes (PATL2, TUBB8, TRIP13, CDC20, TBPL2) have been reported to be the cause of oocyte maturation arrest [4, 18–21]. The patients’ chromosomal analysis indicated a normal female chromosomal complement (46, XX) in this study. However, we did not analysis the genes that caused oocyte maturation arrest. Perhaps there were two different genetic backgrounds which effect on MI oocytes block result in two types of MI-arrest oocytes according to ZP.

The human ZP is the protective coat of the oocyte. It is an extracellular glycoprotein matrix that surrounds the oocyte with a paracrystalline three-dimensional network structure. It serves several functions, such as to mediate sperm binding, induce the acrosomal reaction, prevent polyspermy and protect the embryo until implantation. Zona pellucida gene mRNA expression in human oocytes is related to oocyte maturity, zona inner layer retardance and fertilization competence [22]. The zona pellucida (ZP) of these MI mature-resistant oocytes was observed to be immature, narrow and fibrous [3]. Aberrant spindle structures with the absence of microtubules dispersion of the female chromosomes may be responsible for recurrent human MI oocyte arrest [3, 5]. Recurrent human MI oocyte arrest in owing to highly abnormal spindles, the chances of obtaining normal meiotic progression or even normal fertilization and subsequent embryo development, are very low [5]. It is similar with the smooth ZP oocytes.

However, there were no studies comparing the outcomes of two kinds of recurrent MI-arrest oocytes based on differences of ZP (smooth ZP group and indented ZP group). Oocyte corona complex (OCCC) of both two groups was small and tight, perivitelline space was narrow. All of these oocytes showed the absence of resistance to oolemma penetration during microinjection, and low ooplasm viscosity during aspiration. The difference between two groups: MI oocytes cannot be fertilized in smooth ZP group, whereas MI oocytes with indented ZP can be normally fertilized and develop blastocyst. Patient with indented ZP may had MII oocytes in repeat cycles; Sousa M [7] reported that one patient all oocytes were MI oocytes presenting an indented ZP in the first two cycles. The consequence cycles had MII oocytes. The reason probably differs hormonal stimulation of COS in difference cycles. Another reason may be because of narrow perivitelline space in all oocytes with indented ZP, the polar body was usually difficult to differentiate, and the MII oocytes rate may be not conclusive [6, 7]. Due to abnormal ZP in the indented ZP group, we attempted to use AH before embryo transfer and one patient got pregnant. However, all MI oocytes of smooth ZP group were MI oocytes in all cycles [5].

According to ZP, these two types of MI-arrest oocytes can be used to predict the outcome of recurrent MI oocytes. ICSI combined with assisted oocyte activation (AOA) resulted in no fertilization in MI oocytes with smooth ZP [23]. Given the highly abnormal MI spindle formations and the lack of potential to overcome MI arrest, more research is necessary to reveal the cause of these meiotic arrests. At present, the use of donor oocytes is the only option available for these women with oocyte maturation failure of smooth ZP [1, 4].

Spindle observation and preimplantation genetic diagnosis of MI oocyte embryos can be further investigated.

## Data Availability

Data was available in our computer net.
